# Advanced Speckle-Tracking Echocardiography Could Play a Crucial Role in the Diagnosis of Post-Implanted Cardiomyopathy Associated with a Leadless Pacemaker System

**DOI:** 10.3390/jcm13247692

**Published:** 2024-12-17

**Authors:** Elżbieta Wabich, Ludmiła Daniłowicz-Szymanowicz, Szymon Budrejko, Anna Kochańska, Dariusz Kozłowski, Maciej Kempa

**Affiliations:** 1 Department of Cardiology and Electrotherapy, Faculty of Medicine, Medical University of Gdansk, 80-210 Gdansk, Poland; wabich.ela@gmail.com (E.W.); budrejko@gumed.edu.pl (S.B.); dkozl@gumed.edu.pl (D.K.); kempa@gumed.edu.pl (M.K.); 2 II Department of Cardiology and Electrotherapy, University Clinical Center, 80-952 Gdansk, Poland; akoch@gumed.edu.pl

**Keywords:** speckle-tracking echocardiography, leadless pacing, pacing-induced cardiomyopathy

## Abstract

**Background:** A leadless pacemaker (LP) is a modern alternative to a transvenous pacemaker, allowing certain complications to be avoided; however, some cannot be eliminated. **Aim:** To highlight the essential role of advanced speckle-tracking echocardiography (STE) in diagnosing pacing-induced cardiomyopathy (PICM) caused by an LP. **Clinical case:** A 79-year-old male, after LP implantation a year earlier, was admitted due to heart failure (HF). Left ventricular ejection fraction (LVEF) was 40%, global longitudinal strain (GLS) was −10%, and interventricular mechanical delay (IVMD) was 42 ms. All these parameters were significantly better before the operation. Myocardial work indices confirmed dyssynchrony due to the right ventricular (RV) stimulation pattern, and PICM was considered. To verify the impact of RV pacing on the LV, measurements were taken after restoring the native rhythm, showing an improvement in LVEF (45%), GLS (−13%), IVMD (7 ms), and myocardial work indices. After the next HF exacerbation with further deterioration of the LV function, a decision to convert the LP to a standard CRT-P system was made, with immediate relief in clinical symptoms and improved echocardiographic parameters. **Conclusions:** This case highlights the essential role of STE echocardiography in identifying the detrimental impact of RV pacing, diagnosing PICM, and selecting the appropriate treatment for patients with LPs.

## 1. Introduction

Routinely implanted transvenous pacemakers (TVPs) incorporate electrodes inserted into the chambers of the right heart [1,2]. In some clinical situations, this could lead to further complications, such as vein occlusion related to the leads, tricuspid regurgitation due to valve damage, breakage of the electrodes, and system infections. Pocket infections and cardiac device-related infective endocarditis, usually demanding extraction and later reimplantation, are the most severe and complex problems related to TVPs, worsening the prognosis [3].

Leadless pacemakers (LPs), as an excellent alternative to conventional TVPs, are a modern technique that avoids some of the abovementioned complications. Based on the latest European and national guidelines [4,5], LPs may be recommended for patients without upper extremity venous access, for patients having a high risk of device-related infections or undergoing hemodialysis in whom standard TVPs implantation may be contraindicated. Furthermore, LPs could be implanted as an alternative to standard transvenous VVI systems in the elderly population, given their expected lifespan, to reduce the risk of complications [4]. Considering the abovementioned, LPs became an excellent alternative for patients in whom TVP implantation would be contraindicated or carry a high risk of infective endocarditis or venous thrombosis events. While LPs reduce the risk of some complications, some are impossible to omit. Pacing-induced cardiomyopathy (PICM) seems to be one of those problems.

Typical traditional localization of the ventricular electrode in the right ventricular (RV) apex leads to mechanical dyssynchrony between the RV and the left ventricle (LV). In some patients, that situation could cause a reduction in LV ejection fraction (LVEF) and the development of heart failure (HF) symptoms [2,6] defined as PICM. PICM is diagnosed in patients with HF symptoms, a decrease in LVEF of 10% or more, or below 50% [7]. PICM most often develops within 2–3 years after TVP implantation, with an average of 15 months [8,9].

PICM diagnosis requires immediate HF treatment. While typical pharmacotherapy alone is usually ineffective in such situations, in some patients, the decision to upgrade standard RV pacing to resynchronization therapy needs to be undertaken [2,6,7,10]. Therefore, the precise diagnosis of PICM, after excluding other possible reasons for HF development, is crucial from the clinical point of view. Advanced technology, including speckle-tracking echocardiography (STE), could be essential to recognize and define abnormal deformations of the LV associated with RV pacing, and state proper diagnosis of PICM occurrence [11,12,13]. Although PICM is described in the literature in patients with TVPs, it is not a well-established issue regarding LPs. Clinical experience with LPs is still insufficient, so we do not have enough data regarding PICM in those patients.

## 2. Case Description

**Figure 1:**
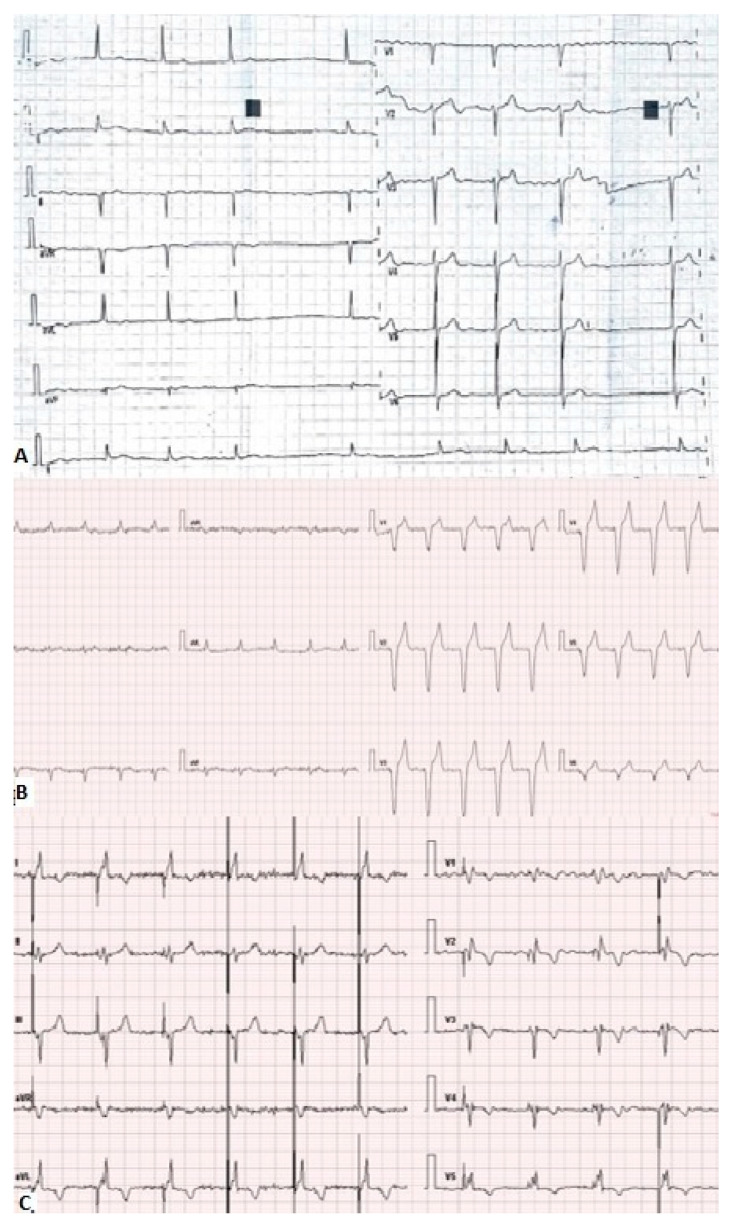
The presentation of the patients’ ECG before LP implantation—QRS duration 100 ms (**A**), after LP implantation with VVI pacing—QRS duration 160 ms (**B**), after CRT-P implantation with biventricular pacing—QRS duration 100 ms (**C**).

We present a case of a 79-year-old male patient who was admitted to our department due to a gradual deterioration of the clinical condition, shortness of breath on minimal exertion and at rest, and dizziness during exercise with an inconclusive decline in exercise capacity since half a year before. One year before, the patient had LP implantation due to permanent atrial fibrillation (AF) with symptomatic bradycardia (average rhythm of 50 beats per minute and QRS duration of 100 ms without any intraventricular conduction disturbances—Figure 1A) and high risk for TVP (medical history of numerous thromboembolic episodes and chronic kidney failure). Additionally, the patient had a history of myocardial infarction (MI) decades ago.

The physical examination at admission revealed swelling of the lower limbs up to the knees and crackles at the base of the lungs on auscultation. A significant increase in B-type natriuretic peptide (BNP) levels (up to 333 pg/mL) was observed in laboratory tests (Table 1). Computed tomography of the lungs and functional tests ruled out pulmonary causes of dyspnea, including chronic obstructive pulmonary disease and interstitial lung disease.

A precise description of the measured parameters is presented in Table 1. In resting ECG AF with permanent VVI pacing, 70 per minute was noticed (with broad QRS complexes up to 160 ms—Figure 1B).

Echocardiographic examination, which was performed on a GE VIVID E95 (Horten, Norway, with an EchoPAC workstation v204, GE Healthcare), showed significant deterioration of LV function, with LVEF decreased to 40% (Supplementary Material Video S2); therefore, HF was initially diagnosed. In the first instance, coronary angiography was performed, and no hemodynamically significant lesions were revealed, which allowed us to exclude ischemia as the most common mechanism of HF development. The LP interrogation demonstrated appropriate pacing parameters and, notably, an exceptionally high pacing percentage, with 99% of ventricular beats being paced. Similarly, other possible reasons for LV function deterioration were excluded, such as myocarditis (low inflammation markers levels, low troponin level, no medical history of viral infections) or valvular disease. Due to the history of LP implantation one year before the hospitalization, PICM development as the possible reason for HF was considered.

**Figure 2:**
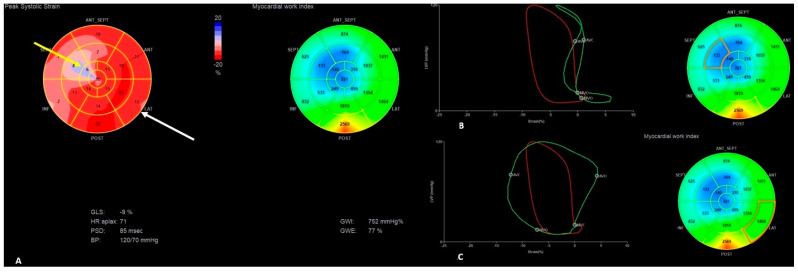
The “Bull’s-eye” representation of longitudinal strain (**A**) and myocardial work index (**B**,**C**) measurements during the first HF exacerbation; the yellow arrow indicates the area characteristic for PICM RV deformation pattern: decreased longitudinal strain and myocardial work indexes in septal middle and apical segments of the LV; the white arrow indicates the excessive myocardial work of lateral wall segments; (**B**) the green chart indicates ineffective myocardial work of the anterior part of the intraventricular septum; (**C**) the green chart indicates excessive myocardial work of the lateral wall segments.

**Figure 3:**
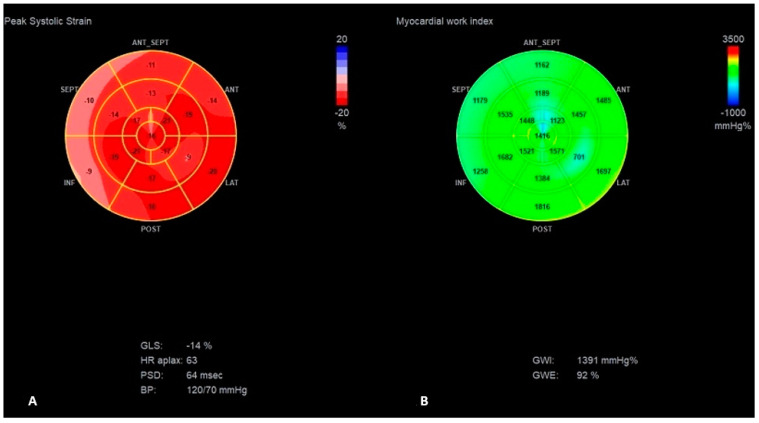
The “Bull’s-eye” representation of longitudinal strain (**A**) and myocardial work index (**B**) measurements before LP implantation.

The presence of apical rocking in a four-chamber apex view (Supplementary Material Video S2), a decrease in global longitudinal strain (GLS) to −10%, and an increase in interventricular mechanical delay (IVMD) to 42 ms were presented (Figure 2), while these parameters were significantly better before LP implantation (LVEF was 55%, GLS was −16%, and IVMD was 10 ms) (Figure 3).

**Figure 4:**
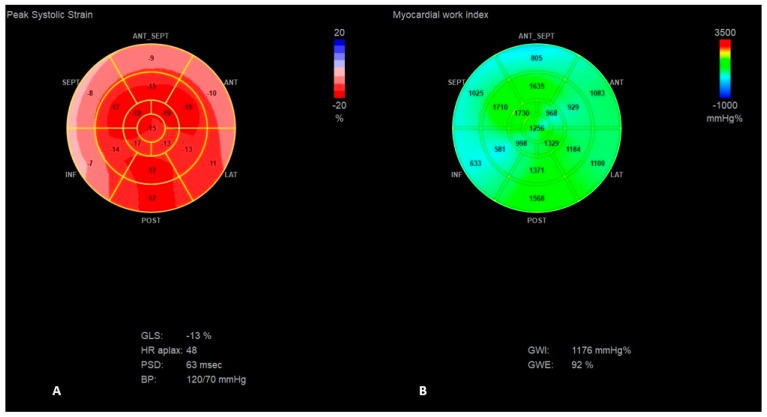
The “Bull’s-eye” representation of longitudinal strain (**A**) and myocardial work index (**B**) measurements during the first HF exacerbation, after switching off VVI pacing.

Hypo-/akinesia of the apex segment of the intraventricular septum (IVS) as the possible consequence of the old myocardial infarction was noticed in the present and previous echocardiography (Supplementary Material Videos S1 and S2). PICM was suspected as the most probable cause of HF; therefore, myocardial work indices using STE were also measured (frame rate of 59 per second). That revealed a pattern typical for RV pacing: disturbed deformation and ineffective work index of the septal segments with prominently increased myocardial work indexes for the lateral wall; LV global work efficiency was reduced to 77% (Figure 2). To check the possible influence of the RV pacing on the above-mentioned parameters, RV pacing was temporarily switched off and the native rhythm (40–48/min) was obtained. This revealed a slight improvement in the LV contractility (LVEF increased to 45% and GLS to −13%) with a shortening of IVMD to 7 ms, and a significant improvement in the work index of the IVS in the above-mentioned segments, along with an improvement in the global efficiency of LV work to 92% (Table 2, Figure 4).

**Figure 5:**
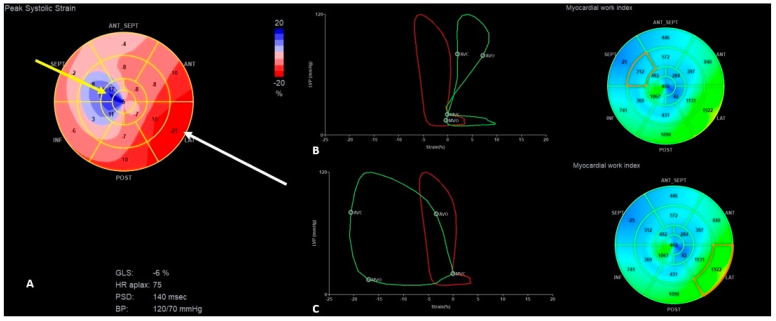
The “Bull’s-eye” representation of longitudinal strain (**A**) and myocardial work index (**B**,**C**) measurements during the second HF exacerbation; the yellow arrow indicates the area characteristic for PICM RV deformation pattern: decreased longitudinal strain and myocardial work indexes in septal middle and apical segments of the LV; the white arrow indicates the excessive myocardial work of lateral wall segments; (**B**) the green chart indicates ineffective myocardial work of the anterior part of the intraventricular septum; (**C**) the green chart indicates excessive myocardial work of the lateral wall segments.

That proved the adverse effect of RV pacing on the LV function. In the first instance, we decided to implement the complete HF pharmacological treatment, which included sacubitril/valsartan, beta-blocker, eplerenone, and dapagliflozin in maximum tolerated doses according to ESC guidelines [14]. Additionally, the pacing rate was lowered twice to 60 bpm and then to 50 bpm to reduce the high pacing percentage. Unfortunately, both attempts were unsuccessful, as Holter monitoring consistently showed a persistently high pacing percentage after each adjustment. Unfortunately, half a year later, the patient was admitted to our department due to attenuation of HF symptoms and a further increase in BNP level (Table 1). Once more, we performed subsequent echocardiography (Supplementary Material Video S3), which revealed further deterioration of LV function (LVEF decreased to 34%). The STE analysis, compared to the previous examination, confirmed further intensification of dyssynchrony in the strain pattern (GLS −6% vs. −11% without pacing, LV work efficiency 79% vs. 85% without pacing), and worsening of general contractility (LVEF during VVI pacing was 34%, while without pacing it was 45%), which is presented in Figure 5. All echocardiographic parameters are presented in Table 2.

**Figure 6:**
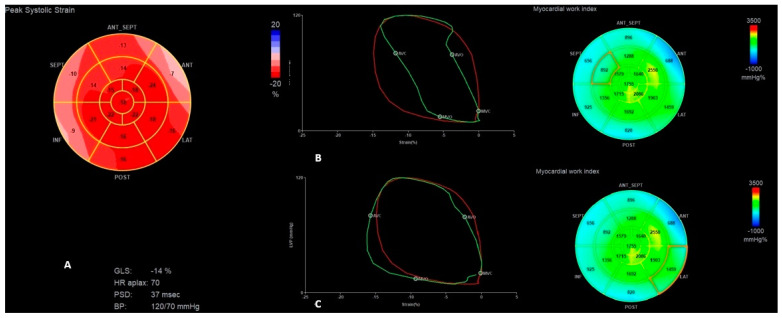
The “Bull’s-eye” representation of longitudinal strain (**A**) and myocardial work index (**B**,**C**) measurements after CRT-P implantation with a significant improvement in septal segmental strain and MWI values. (**B**,**C**) presents the significant improvement in myocardial work values for intraventricular (**B**) and lateral (**C**) segments.

Finally, considering the worsening of LV function due to RV pacing, despite the patient’s potential risk for TVP implantation, the CRT-P system (with transvenous RV and LV leads, and without an atrial lead due to the chronic atrial fibrillation) was implanted to minimize cardiac contraction dyssynchrony. On the third day after CRT-P implantation, a significant improvement in LV contractility was documented: complete elimination of ventricular dyssynchrony (IVMD 3 ms) and significant improvement in general deformation (GLS −15%, LVEF 45%) (Figure 6, Supplementary Material Video S4).

**Figure 7:**
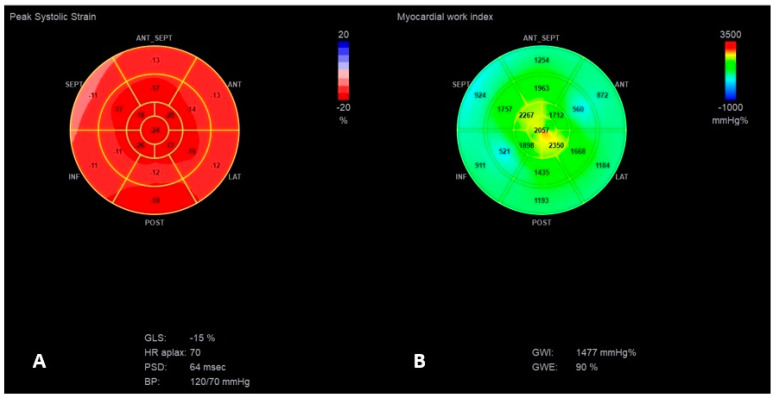
The “Bull’s-eye” representation of longitudinal strain (**A**) and myocardial work index (**B**) measurements at 6-months follow-up.

The resting ECG revealed significant reduction in the duration of QRS complexes to 100 ms (Figure 1C). The patient declared immediate relief in clinical symptoms. At 6-months follow-up, the patient presented the same clinical status, with stable echocardiographic parameters (Table 1 and Table 2, Figure 7). The patient’s condition remains stable, with a continued marked improvement in symptoms until 6 December 2024.

## 3. Discussion

The phenomenon of PICM, although not frequent, is well known regarding TVPs [7,10] but not well established for modern LPs. Some previous studies have identified factors that could predict PICM: male gender, history of MI, AF, chronic kidney disease, QRS widening before implantation, paced QRS duration, and the RV pacing percentage [8,9,10,15,16]. However, the LV function before pacemaker implantation is the most important factor [15,16] in predicting PICM due to unphysiological RV stimulation. According to the present guidelines, LVEF below 50% is the only crucial echocardiographic parameter in choosing between standard RV and CRT stimulation [4]. Some of those factors (male gender, AF, MI, chronic kidney disease) were presented in our patient, in addition to hypo-/akinesia of the apex segment of IVS (the area particularly affected by the RV pacing); however, LVEF was higher than mentioned above, and QRS duration was also not prolonged.

Regarding PICM, due to LPs, data are scarce and inconsistent [15,17,18]. Some authors postulate a lower risk for PICM in LPs than TVPs [15], while others report a similar rate of that complication [17,18]. It is well known that septal pacing, compared to apical pacing, causes a lower risk for PICM in TVPs [19], which could be the same for LPs [20,21] according to the Shantha G. et al. study. However, in our patient, the LP was initially placed in the septal position, which, according to the current literature, is believed to lower the risk of non-physiological stimulation pathways and the subsequent onset of PICM. The patient’s case only confirms that the exact mechanism of PICM development in the LP group remains unclear.

Additionally, since there is a need for proper literature evidence, it is not known whether it differs from the mechanisms described for standard PMs. Saeed Al-Asad et al. propose that the degree of RV pacing alone is not the only predictor of PICM [18]. They also highlight that variations between LP and TVP can affect LVEF in different ways, playing a role in the development of PICM [18].

Echocardiography using modern technologies, particularly on STE and myocardial work calculations, seems crucial in PICM diagnosis [11], as documented in the presented case. There are several reports regarding the use of STE in predicting the development and diagnosis of PICM; however, all are dedicated to TVPs. [11,12,13]. For instance, Ahmed FZ. et al. demonstrated that assessing GLS one month post-pacemaker implantation reliably predicted pacing-induced LV dysfunction after one year [19]. Jung Yeon Chin MD et al. proved that PICM was relatively more common in patients with an LV GLS below −20.7% before implantation [13], which could suggest GLS as a risk factor for PICM development. Data from the literature proves that STE could be helpful in diagnosing PICM and predicting the occurrence of this phenomenon in patients even before pacemaker implantation [11,12,13,19]. That could apply to our patients.

In our patients, we used the noninvasive myocardial work index analysis as an interesting and novel echocardiographic tool that integrates LV strain measurements with an estimated LV pressure [22]. Unlike measuring strain values alone, assessing myocardial work indexes takes afterload into account, a key advantage in the diagnostic process [22]. As we presented in our patient, dyssynchronous contraction causes changes in myocardial work distribution, with lower myocardial work values in the early-activated intraventricular septum and increased myocardial work values in the late-activated lateral wall (Figure 2 and Figure 5). According to the literature, more significant differences between the lateral wall and septum are associated with more pronounced reverse remodeling after cardiac resynchronization therapy [23,24]. In the presented case, proper diagnosis of PICM due to the RV pacing in LP was possible based on a thorough analysis of the coincidence between the time of LP implantation, symptoms onset, and echocardiographic measurements, including myocardial work calculations, confirming a typical RV pacing pattern.

Our decision to convert an LP to an intravenous pacemaker was difficult. That was due to a number of reasons. Firstly, no dedicated removal systems are available for use in our patient LP system. Secondly, upgrading an LP to a CRT device means converting to a standard TVP, which we initially wanted to avoid. There are only two cases regarding an LP upgrade to a resynchronization system due to PICM development described in the available literature: the first described the unconventional upgrade using two sinus coronary leads [25], and the second was with the left bundle branch area pacing [26]. However, these are still non-standard solutions resulting more from clinical experience than established research, and there are no clear recommendations on this topic in the current guidelines.

## 4. Study Limitations

This study focuses on a single case, and its findings cannot be broadly extrapolated. Since LP is a cutting-edge approach in electrotherapy, only a small fraction of patients currently have LP implanted, while the majority continue to use standard TVP systems. The number of studies on LP populations remains limited, with only a few reports addressing the occurrence of PICM in this group. Further research involving larger cohorts of patients with LP pacemakers is necessary to gain deeper insights into the development of PICM in this population.

## 5. Conclusions

Our case presents the crucial role of STE in revealing the harmful effect of RV pacing, PICM diagnosis, and choosing the appropriate treatment in patients with LP. Further studies are needed to examine the risk factors of PICM development in patients qualified to receive LPs.

## Figures and Tables

**Table 1:** Laboratory tests.

Parameter	Normal Range	Baseline Hospitalization	First HF Exacerbation	Second HF Exacerbation	6 Months Follow-Up
**Hemoglobin (g/dL)**	12–15	16	15.7	15.2	15.9
**BNP (pg/mL)**	<89	138	333	220	212
**hsTnI (ng/mL)**	<0.0156	0.0200	0.011	0.012	0.012
**Potassium (mmol/L)**	3.5–5.1	4.4	5.3	5.5	5.0
**Creatinine (mg/dL)**	0.73–1.18	1.14	1.19	1.22	1.17
**GFR (%)**	70–99	62	58	56	60
**Glucose (mg/dL)**	70–99	92	98	88	93

BNP—Natriuretic peptide B, hsTnI—high sensed troponin I; GFR—glomerular filtration rate.

**Table 2:** Echocardiographic parameters.

Parameter	Baseline Examination	First HF Exacerbation		Second HF Exacerbation		Examination After CRT-P Implantation	
	Intrinsic Rhythm	VVI Pacing	Intrinsic Rhythm	VVI Pacing	Intrinsic Rhythm	BiV VVI Pacing	6 mo. Follow-Up
LVEF (%)	55	40	45	34	45	45	50
IVMD (ms)	10	42	7	52	3	3	5
STE parameters							
GLS (%)	−14	−10	−13	−6	−11	−15	−15
PSD (ms)	63	85	63	140	53	40	61
GWE (%)	92	77	92	79	85	90	89
GWW (mmHg%)	138	414	113	263	162	180	220
GCW (mmHg%)	1757	1405	1446	1044	1235	1825	1866
GWI (mmHg%)	1391	752	1176	613	1241	1441	1436

LVEF—left ventricular ejection fraction, IVMD—intraventricular mechanical delay, GLS—global longitudinal strain, PSD—peak strain dispersion, GWE—global myocardial work efficiency, GWW—global wasted work, GCW—global constructive work, GWI—global myocardial work index.

## Data Availability

Data are available upon request.

## Supplementary Materials

The following supporting information can be downloaded at: https://www.mdpi.com/article/10.3390/jcm13247692/s1. Video S1: The 4-chamber echocardiographic view of LV contractility (LVEF 55%) before VVI LP implantation. Video S2: The 4-chamber echocardiographic view of LV contractility (LVEF 40%) during the first HF exacerbation after VVI LP implantation; visible discrete apical rocking movement, and the septal flash also occurred. Video S3: The 4-chamber echocardiographic view of LV (LVEF 34%) contractility during the second HF exacerbation after VVI LP implantation. Video S4: The 4-chamber echocardiographic view of LV contractility (LVEF 50%) 6 months after CRT-P implantation.
